# Novel Synthetic Peptide Agelaia-12 Has Improved Activity Against *Mycobacterium abscessus* Complex

**DOI:** 10.3390/pathogens13110994

**Published:** 2024-11-13

**Authors:** Arthur Alves Coelho, Lília Cristina de Souza Barbosa, Adeliane Castro da Costa, André Kipnis, Ana Paula Junqueira-Kipnis

**Affiliations:** 1 Department of Biosciences and Technology, Tropical Pathology and Public Health Institute, Federal University of Goiás, Goiânia 74605-050, GO, Brazil; arthur.avcoelho@gmail.com (A.A.C.); lcristinasb19@ufg.br (L.C.d.S.B.); adeliane.costa@ufj.edu.br (A.C.d.C.); akipnis@ufg.br (A.K.); 2 Health Sciences Academic Unit, Federal University of Jataí, Jatái 75801-615, GO, Brazil

**Keywords:** antimicrobial, resistance, nontuberculous mycobacteria

## Abstract

Fast-growing mycobacteria cause difficult-to-treat infections due to their high intrinsic resistance to antibiotics as well as disinfectant agents. *Mycobacterium abscessus* complex (MAC) is the main cause of nontuberculous mycobacteria diseases. In this work, we evaluated the activity of the novel synthetic antimicrobial peptide, Agelaia-12, against *Mycobacterium abscessus* and *M. massiliense*. Agelaia-12 showed a minimum inhibitory concentration (MIC) of 25 μM detected against *M. abscessus* and *M. massiliense* with no cytotoxicity. The scanning electronic microscopy analysis of mycobacterial treated with Agelaia-12 demonstrated the presence of filamentous structures and aggregation of the cells. Congo red binding assay of *M. abscessus* exhibited altered dye accumulation after treatment with Agelaia-12. Treatment of *M. abscessus*- or *M. massiliense*-infected murine macrophages with Agelaia-12 decreased the mycobacterial load by 92% for the tested strains. Additionally, IFN-y KO mice infected with *M. abscessus* or *M. massiliense* and treated with Agelaia-12 showed a 98% reduction in lung bacterial load. Thus, the synthetic peptide Agelaia-12 may be a promising biomolecule for the treatment of mycobacteriosis, and its structural properties may serve as a foundational model for the design and development of novel pharmaceutical agents aimed at combating this disease.

## 1. Introduction

The *Mycobacterium abscessus* complex (MABC), also known as nontuberculous mycobacteria (NTM), is a type of pathogenic mycobacteria that frequently presents drug [1] and disinfectant resistance [2]. This complex is divided into three subspecies: *Mycobacterium abscessus* subsp. *abscessus* (*M. abscessus*), *Mycobacterium abscessus* subsp. *massiliense* (*M. massiliense*), and *Mycobacterium abscessus* subsp. *bolletii* (*M. bolletii*) [3]. They are a group of environmental bacteria widely found in water and soil resources, as well as inside amoeboid organisms and in biofilms present in pipes and liquid reservoirs [3,4,5].

The MABC has stood out for its growing number of outbreaks of infections worldwide [6], especially healthcare-associated infections (HAIs) [7,8]. It can establish opportunistic infections in immunocompromised, immunocompetent, traumatized, and post-surgical individuals [5,9]. MABC infection occurs mainly in the lungs, skin, and soft tissues, but it can also affect other organs, such as the eyes and central nervous system [4,10]. *Mycobacterium abscessus* infections are phenotypically diverse and manifest as a broad spectrum of chronic diseases associated with individuals with immunosuppression and/or underlying lung diseases such as cystic fibrosis, non-cystic bronchiectasis, bronchiolitis, chronic obstructive pulmonary disease, and previous tuberculosis. The diagnosis includes clinical, radiographic, histopathological, and microbiological examinations to determine prognosis and treatment [11].

The American Thoracic Society/Infectious Diseases Society of America recommends treatment for MABC infections with the associated use of macrolides, aminoglycosides, beta-lactams, tetracyclines, carbapenems, oxazolidinones, riminophenazines, and/or cephamycins [12]. However, treatment for infections by MABC bacteria is challenging, as it includes an extensive regimen of intravenous and oral antimicrobial administration to overcome the acquired resistance of these bacteria to some of these antibiotics [13,14]. Thus, drug resistance limits therapeutic options, with few effective active antibiotics available. Generally, for the treatment of infections caused by *M. massiliense*, clarithromycin is the main antibiotic used [15], although studies have shown that some strains of this subspecies have shown resistance to this drug [16]. The pattern of resistance differs among the three subspecies of the MABC, in which *M. abscessus* and *M. boletti* are generally resistant to macrolides, while *M. massiliense* is sensitive to this class of antibiotics [17,18] and resistant to fluoroquinolones [19]. In bacteria of this complex, the molecular mechanism of resistance involves enzymes that promote the modification of antibiotics’ target [20] such as the expression of a ribosome methylase encoded by the erm and rrs genes that result in resistance to macrolides and aminoglycosides, respectively, or mutations in the gene rrl 23S RNA [21]. Prolonged multimicrobial antibiotic therapy for the treatment of drug-resistant MABC strains leads to drug-induced toxicity, which leads to treatment discontinuation, patient non-compliance [22], and high rates of recurrent or chronic infections and severe clinical outcomes [23]. Thus, the multidrug resistance and limited therapeutic options available for the treatment of MABC infections reinforce the importance to develop new drugs [20].

Antimicrobial peptides (AMPs) are short sequences of amino acids (less than 100 amino acids) that occur naturally as part of the host defense system of all domains of life [24,25], present mainly in the venom of invertebrates, such as bees [26,27], scorpions [28,29,30], and wasps [31,32,33]. AMPs have different classes according to their structural composition, including amphiphilic α-helices, glycopeptides, lipopeptides, and short cationic peptides [30,31], which determine their mechanism of action and antimicrobial activity. AMPs bind to the membranes of microorganisms including mycobacteria [34] by electrostatic bonds, making them more permeable, inhibiting wall biosynthesis, ands affecting lipid metabolism, which could culminate in cell lysis [35]. Thus, AMPs are promising candidate molecules as antimicrobial agents for the treatment of infections caused by microorganisms [36]. Our research group has demonstrated the antimicrobial therapeutic activity of invertebrate-derived peptides. Studies with NDBP-5.5 [37] and ToAP2 [38] derived from scorpion venom and Polydim-I [39] and Polybia-MPII [40] derived from wasp venom showed bactericidal activity against *M. masiliense.* Among the various classes of AMPs that exist, mastoparans are peptides found in the venom of social and solitary wasps with cationic and amphipathic α-helical structures, capable of promoting mast cell degranulation and lysis action of prokaryotic and eukaryotic cell membranes [41,42], thus enabling cell penetration, with toxigenic, cytotoxic, or antimicrobial activities [43]. Agelaia-MPI, a 14-residue-long peptide isolated from the wasp venom of *Parachartegurs fraternus*, is one example of a mastoparan AMP that has already demonstrated potential as a broad antimicrobial agent due to its cytolytic and cationic properties that favored cell envelope and membrane disturbance [36]. Since the most common resistance mechanisms of *M. abscessus* are from expression of antibiotic-target-modifying enzymes or efflux pumps, targeting membrane/membrane-like disruption is a promising therapeutic strategy (revised by [1,44]). From this perspective, we hypothesized that Agelaia-MPI-derived AMPs could have antimicrobial action against multidrug-resistant bacteria and thus we designed novel sequences, Agelaia-KK and Agelaia-12, combining enhanced cationicity and amphipathicity but probably less cytotoxicity to eukaryotic cells [45].

In this context, this research aimed to study if the novel antimicrobial peptide Agelaia-12 presents antimicrobial activity against *Mycobacterium abscessus* complex bacteria.

## 2. Materials and Methods

### 2.1. In Silico Structural Analysis of the Antimicrobial Peptide Agelaia-12

The synthetic peptide Agelaia-12 was synthesized by Aminotech Development and Technology (São Paulo, Brazil) using the solid-phase chromatography strategy and purified by RP-HPLC (≥95%). The multiple alignment between the sequence of the natural peptide MPI and the synthetic peptides Agelaia-KK and Agelaia-12 was performed using the Clustal Omega software version 2.0 (https://www.ebi.ac.uk/jdispatcher/msa/clustalo, accessed on 15 March 2024). The formation of a helical wheel to observe the conformation of charged residues within the alpha helix was generated by the HeliQuest software version 2.0 (https://heliquest.ipmc.cnrs.fr/index.html, accessed on 1 February 2024) and the prediction of physicochemical parameters was determined through the digital tool Expansy ProtParam version 1.0 (https://www.expasy.org/resources/protparam, accessed on 12 March 2024). The prediction of polar and nonpolar surfaces was generated by the online tool Aggrescan 3D version 2.0 (https://biocomp.chem.uw.edu.pl/A3D2/, accessed on 2 April 2024). The prediction of the secondary structure was performed in I-TASSER software (https://zhanggroup.org/I-TASSER/, accessed on 1 October 2024), based on the threading methodology [46].

### 2.2. In Vitro Antimicrobial Activity Assessmentv

In vitro evaluation of antimycobacterial activity of the peptide Agelaia-12 was performed using an inhibition assay by broth microdilution in a 96-well plate, against strains of *M. massiliense* (GO06) or *M. abscessus* (ATCC 19977) at 10^5^ CFU/mL. The strains were obtained from the Brazilian culture collection of the IPTSP (https://rgptb.iptsp.ufg.br/p/35018-colecao-de-cepas-de-m-abscessus-subsp-massiliense, accessed on 1 January 2020) of the Laboratory of Molecular Bacteriology of the IPTSP. The samples were reactivated in Luria–Bertani (LB) medium, and evaluation was carried out by microscopic observation of slides containing stained smears using the Ziehl–Neelsen coloration. Each Agelaia-12 dilution assay was performed in triplicate, in which different concentrations of the peptide were evaluated, from 200 μM to 6.25 μM, and compared with the action of amikacin at 54.6 μM used as the inhibition control. After 48 h of incubation, 30 μL of resazurin was added to each well (resazurin is an indicator of cell metabolism, which has a blue color, and in the presence of cell metabolic activity, it is reduced to resopurine, a molecule that has a pink color), allowing the observation of antimicrobial activity [47] and the visual determination of the minimum inhibitory concentration (MIC) of the peptide under evaluation. The assays to determine the MIC were repeated twice with three biological replicates.

In order to evaluate if Agelaia-12 presented bactericidal or bacteriostatic activities, *M. massiliense* (GO06) or *M. abscessus* (ATCC 19977) at 10^5^ CFU/mL were cultivated and treated as described above. After this period, serial dilutions were made in PBS-Tween 80 0.05% (PBST) and the bacterial suspensions were seeded in LB agar to determine the colony-forming units (CFUs). The percentage of bacterial growth inhibition was determined by comparison to the CFUs counted for each strain grown in LB agar alone.

### 2.3. Analysis of the Effect of Agelaia-12 Peptide on M. abscessus by Scanning Electron Microscopy

To verify whether Agelaia-12 promotes structural changes on the cell surface of *M. abscessus* ATCC, scanning electron microscopy (SEM) analyses were performed according to [48] with some modifications. The strains were cultivated on Mueller–Hinton agar medium for 5 days at 35 °C. After the incubation period, the colonies were isolated by cutting agar fragments and placing them in 48-well plate wells. In each of these wells, 300 μL of Agelaia-12 (50 μM) diluted in MH medium was pipetted, and for the positive control for bacterial growth, the same volume of Muller–Hinton liquid medium was pipetted. The plate was incubated at 35 °C for 24 h. Then, the Agelaia-12 solution was carefully removed from each well and the samples were fixed, adding 300 μL of Karnovsky solution (2% paraformaldehyde, 2 glutaraldehyde, 0.01 M sodium cacodylate buffer) at 4 °C for 30 min. After that, the samples were dehydrated in an increasing ethyl series (ethyl alcohol 30%, 50%, 70%, 90%, and 100%) for 10 min each pass, followed by acetone and hexamethyldisilazane (HMDS) (*v*/*v*) and then in pure HMDS for 5 min each. The agar fragments containing the colonies were removed from the plate, glued to stubs with the aid of double-sided tape, metallized in gold (Dentum Vacuum Desk V metallizing device), and analyzed in a scanning electron microscope (model JSM—6610, Jeol, Tokyo, Japan, Thermo scientific NSS Spectral Imaging) at the Multiuser Laboratory of High-Resolution Microscopy, LabMic, UFG, Goiânia, Brazil.

### 2.4. Analysis of the Effect of Agelaia-12 Peptide on the Surface of M. abscessus by Congo Red Staining

Mycobacteria cell surface integrity was investigated by Congo red cell staining according to [48], with slight modifications. *M*. *abscessus* ATCC 19977 was cultured on LB broth medium for 5 days at 37 °C, then the cells were recovered, and the culture was adjusted to 1 OD at 590 nm in a final volume of 3 mL of LB broth medium with Tween 80 (0.05%) and Congo red 1% (*v*/*v*). Each tube received either Agelaia-12 (50 μM) or polymyxin B (3.8 μM). LB medium with Tween 80 (0.05%) was used as a control for bacterial growth. After 48 h of incubation at 37 °C under agitation, the samples were centrifuged and washed three times with PBS. An aliquot of each tube was serially diluted and plated on LB agar for CFU determination. The remaining cultures were centrifuged, resuspended on 2 mL of acetone, and homogenized for 2 h. The optical density (OD) of the sample was read at 488 nm. The relative Congo red accumulation in the cells was adjusted by the concentration of cells obtained by CFU determination with the following formula: (ODt ÷ ODc) × (CFUt ÷ CFUc), where ODt is the OD for the treatment; ODc is the OD of the growth control; CFUt is the CFUs counted for the treatment; CFUc is the CFUs counted for the growth control.

### 2.5. Hemolysis Assay

The assay was carried out according to [37], with modifications. Blood from a health donor of the blood bank of the Laboratório Prof. Margarida Dobler Komma (Instituto de Patologia Tropical e Saúde Pública, UFG, Goiânia, Brazil) was used. The red blood cells were obtained by centrifugation at 1000× *g* for 5 min, washed three times with sterile PBS, and the concentration was determined in a hemocytometer (Horiba ABX, Irvine, CA, USA). After adjustment to 1 × 10^8^ cells/mL, 50 μL of the sample was added into 96-well plate wells. The Agelaia-12 peptide was resuspended in PBS at concentrations 16, 8, 4, 2 and 1× that of the MIC (25 μM), and 50 μL was added to the wells containing the red blood cells (*v*/*v*). Red blood cells treated with Triton-100X (1%) were used as the positive control. The negative control consisted of untreated red blood cells. The plate was incubated for 1 h at 37 °C, and after this period, the plate was centrifuged at 1000× *g* for five minutes. Then, the supernatant was removed from each well and pipetted into an empty well for OD readings. Absorbance was determined at 540 nm with a Multiskan SkyHIgh microplate reader (Thermo Fisher Scientific, Waltham, MA, USA). The percentage of hemolysis was obtained from the formula: % hemolysis = 100 × [(test-control negative) ÷ (control positive − control negative)], as described by [37].

### 2.6. Animals

In the in vivo experiments, both female and/or male mice, 6–8 weeks old, IFN-y KO and BALB/c, obtained at the Multiuser Center for Animal Production and Experimentation (CMPEA) at the Institute of Tropical Pathology and Public Health of the Federal University of Goiás (IPTSP/UFG), were used. The animals were kept in a microenvironment with controlled temperature (24 ± 1 °C), humidity (50 ± 1%), and light conditions (12 h of light and 12 h of darkness), in addition to receiving food and water ad libitum throughout the experimental procedure. The study was approved by the Ethics Committee on Animal Use (CEUA, protocol No. 052/22) at UFG.

### 2.7. Culture of Peritoneal Macrophages

Peritoneal macrophages were obtained from female BALB/c mice, 6–8 weeks old, from CMPEA, after a period of 72 h of stimulation by thioglycolate intraperitoneally. After euthanasia, 5 mL of ice-cold PBS was injected into the peritoneal cavity, followed by vigorous intercostal massage. Viable peritoneal cells were counted using trypan blue (code 1263C061, Amresco, Solon, OH, USA 44139-4300), and their concentration was adjusted to 10^6^ cells per mL in complete RPMI medium (cRPMI-1640, Sigma-Aldrich, Gillingham, UK) containing 2 mM glutamine, 100 U/mL penicillin, 1000 U/mL streptomycin (GIBCO), 10 nM pyruvate, and 10% fetal bovine serum (FBS). A total of 2 × 10^5^ cells per well were added to 96-well plates and incubated at 37 °C with 5% CO_2_ for 24 h for adhesion. After the incubation time, the cells were infected with the following strains: *M. abscessus* ATCC 19977 and *M. massiliense* GO06 with a multiplicity of infection (MOI) of 1:1, for a period of 3 h. Then, the cells were washed three times with PBS to remove bacteria from the extracellular medium. The infected cells were treated with the peptide Agelaia-12 at 50 μM and 100 μM, or with amikacin (54.6 μM), under the same incubation conditions mentioned above for 24 h. After treatments, infected macrophages were washed three times with RPMI medium (RPMI-1640, Sigma-Aldrich, UK) and then lysed with water (200 μL), and the suspensions were plated on LB agar medium to determine the CFU load of intracellular bacteria.

### 2.8. Determination of Mycobacterial Load in the Lungs of IFN-y KO Mice Infected with M. abscessus or M. massiliense

For this assay, IFN-y KO mice were used according to the protocol established by [49] with some modifications. The animals were infected intravenously with 100 μL of *M. abscessus* ATCC 19977 (10^6^ CFU/mL) or 100 μL of *M. massiliense* GO06 (10^8^ CFU/mL) [50], and 18 days after infection, the intraperitoneal treatment was initiated. The animals in the treatment group received 0.5 mg/kg of the Agelaia-12 peptide for a period of 7 days, while the control group received intraperitoneal treatment with sterile saline phosphate buffer (PBS) for the same period. At the end of this period, the animals were euthanized, and the lungs were collected in 1 mL of PBST, homogenized, and serially diluted in PBST and plated on LB agar. After five days of incubation at 37 °C, the colonies were counted, and the lung mycobacterial load was determined.

### 2.9. Histopathological Analysis

The right lung caudal lobes of euthanized mice treated or not with 0.5 mg/kg of Agelaia-12 were collected according to the timeline. The lobes were perfused with 10% buffered formaldehyde, and soon after collection, they were stored in identified histological cassettes, fixed in 10% buffered formaldehyde for 24 h, and preserved in 70% alcohol until processing. Histological sections were obtained at 5 μm thickness and stained with hematoxylin and eosin (HE) for light microscope analysis.

### 2.10. Statistical Analysis

The results were tabulated using Google Sheets Online Software (https://workspace.google.com/, accessed on 1 October 2024) and the graphs were plotted with Prism software package (version 8.0, GraphPad, La Jolla, CA, USA, 2019). The normality among results obtained after treating the mice with Agelaia-12 was tested using the Shapiro–Wilk test. The results were nonparametric; thus, the comparison was evaluated using a Mann–Whitney test. Significant differences were considered when *p* < 0.05. All repetitions of the experiments showed similar responses.

## 3. Results

### 3.1. Bioinformatic Studies of Agelaia-12 Structural Characteristics

**Figure 1:**
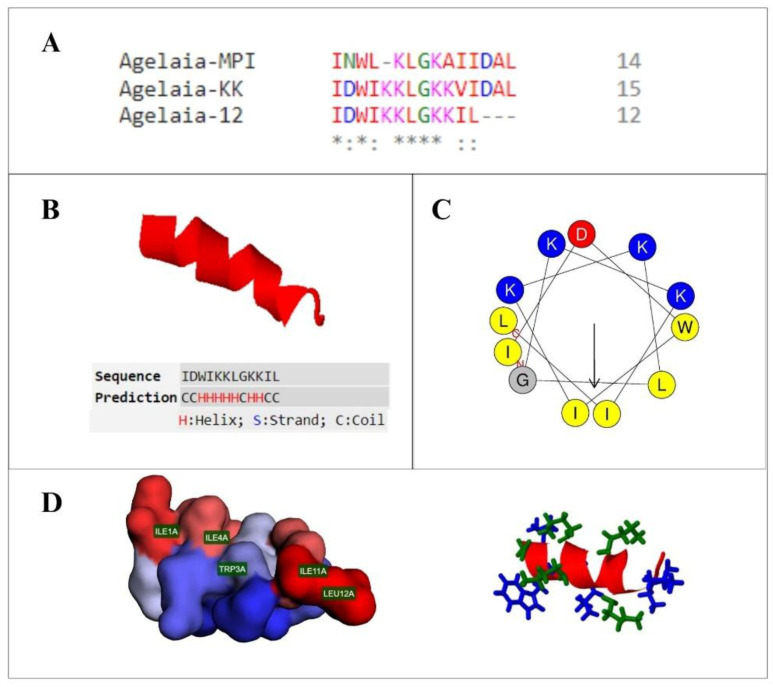
Structure of the antimicrobial peptide Agelaia-12. (**A**) Multiple alignment of the natural peptide Agelaia-MPI and derived synthetic peptides, Agelaia-KK and Agelaia-12. (*) identical amino acids, conserved region and (:) semiconservative amino acids substitutions. (**B**) Three-dimensional secondary structure of the peptide Agelaia-12, demonstrating its predominant alpha helix secondary structure. (**C**) Diagram of the helical wheel of the Agelaia-12 peptide, with a region composed of polar and cationic residues, in blue, and nonpolar residues, which are mostly hydrophobic, in yellow. The arrow indicates the direction of the hydrophobic moment of the molecule. (**D**) On the left, a representation of the polar (in blue) and nonpolar (in red) surfaces of the peptide is demonstrated, highlighting the location of nonpolar residues, and on the right side, a representation of the side chains of charged (green) and uncharged (blue) residues.

The artificial sequence of the Agelaia-12 peptide was rationally derived from the mastoparan peptide Agelaia-MPI (BRASIL, 2022—patent filed, [45]) by inserting an aspartate residue and two lysine residues and removing three terminal amino acids, as demonstrated in the multiple alignment (Figure 1A). After the proposed modifications, the peptide structure predicted by the threading methodology revealed a secondary alpha helix structure, as illustrated in Figure 1B. The C-score obtained for this model was −0.5, allowing us to attest to the reliability of the prediction (C-score > −1.5) and the alpha helix conformation of the molecule [51]. Furthermore, from the observation of the helical wheel, it is possible to infer the amphiphilic conformation of the peptide, endowed with a surface composed of polar and cationic residues and nonpolar residues, which are mostly hydrophobic (Figure 1C,D). The general physicochemical properties of the peptide are shown in Table 1, highlighting the total positive charge of the peptide as +3 and its relatively high hydrophobicity value.

### 3.2. Agelaia-12 Inhibits M. abscessus spp. Growth

**Figure 2:**
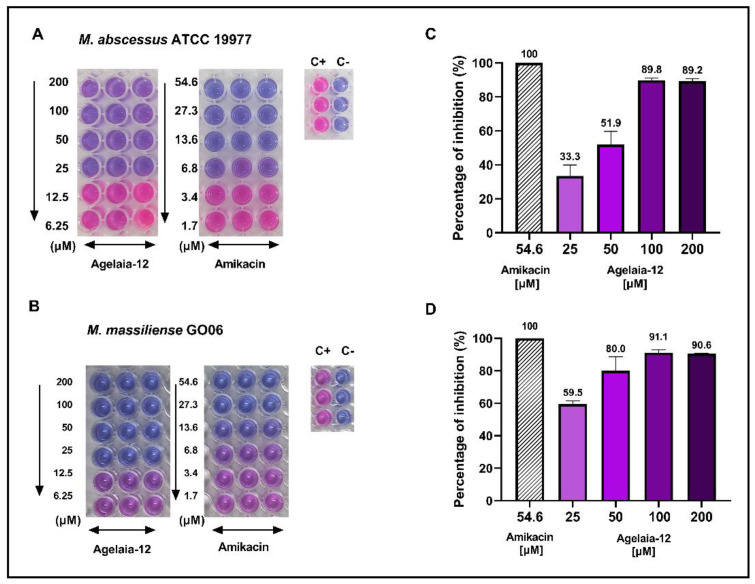
Evaluation of in vitro antimicrobial activity of Agelaia-12 against *M. abscessus* and *M. massiliense strains.* Cell culture plates demonstrating the antimicrobial microdilution inhibition assay with decreasing concentrations of Agelaia-12 and amikacin (used as a control for inhibition) after the addition of resazurin against *M. abscessus* (**A**) and *M. massiliense* (**B**). Acronyms: C+: control of bacterial growth; C-: control of the absence of bacterial growth. Percentage of bactericidal activity determined after plating the wells incubated in similar conditions of resazurin assay with different Agelaia-12 treatments and amikacin (54.6 µM) for *M. abscessus* ATCC (**C**) and *M. massiliense* GO06 (**D**).

The MIC was defined as the lowest concentration of the antimicrobial that inhibited bacterial growth in vitro, which was visually verified by the colorimetric change in the metabolic indicator resazurin [52]. The MIC of Agelaia-12 was 25 μM for *M. abscessus* (Figure 2A) and *M. massiliense* (Figure 2B). To investigate whether the inhibition was bactericidal or bacteriostatic after incubation with AMPs, cultures were plated on LB agar (Figure 2C,D). Although the MIC was 25 μM, at this concentration, 40–60% of bacteria remained viable. The minimal bactericidal concentration (MBC) was not achieved at a concentration as high as 200 μM for both strains. Since MBC is more than eight times greater than MIC, Agelaia-12 has a bacteriostatic activity.

### 3.3. Treatment with Agelaia-12 Impairs the Morphological Characteristics of M. abscessus

**Figure 3:**
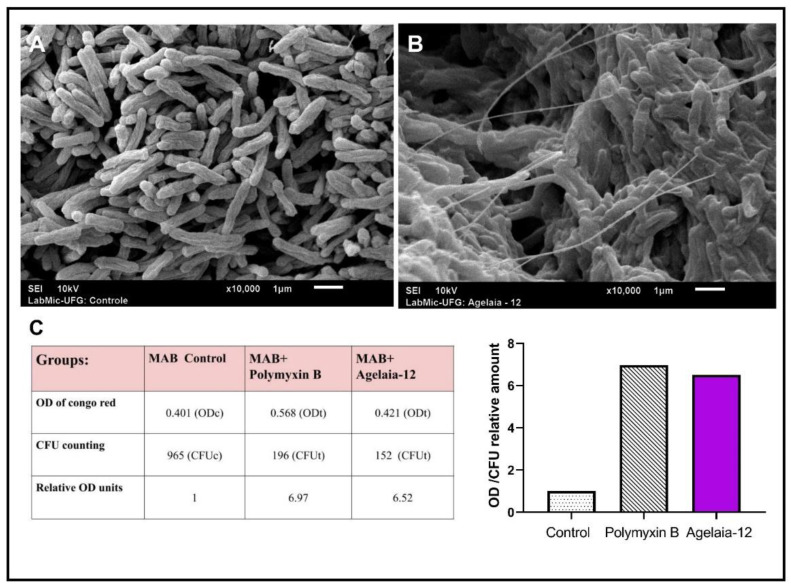
SEM analysis of *M. abscessus* and surface binding of Congo red. (**A**) SEM analysis of nontreated *M. abscessus* colonies. (**B**) *M. abscessus* colonies treated with Agelaia-12 (50 μM) showing aggregation and filamentous-like structures. (**C**) Data obtained from Congo red assay. *M. abscessus* treated with Agelaia-12 or polymyxin had increased retention of the dye (OD of supernatant) with inhibited growth (CFU count). The graph on the right represents the adjusted Congo red dye retention for each treatment.

Once Agelaia-12 apparently presented antimicrobial function (inhibition of 60 to 85%) against *M. abscessus*, it was evaluated if this AMP was able to modify its morphology. Figure 3 shows the morphological characteristics of *M. abscessus* when untreated (Figure 3A) and treated with Agelaia-12 (Figure 3B), using SEM. It was observed that the treatment with Agelaia-12 led to the accumulation of filaments on the cells of *M. abscessus* as well the induction of bacterial aggregation. The Congo red binding assay demonstrated the capacity of Agelaia-12 to disturb the surface of *M. abscessus*, similarly to the drug polymyxin B control (Figure 3C).

### 3.4. Absence of Red Blood Cell Toxicity of Agelaia-12

**Figure 4:**
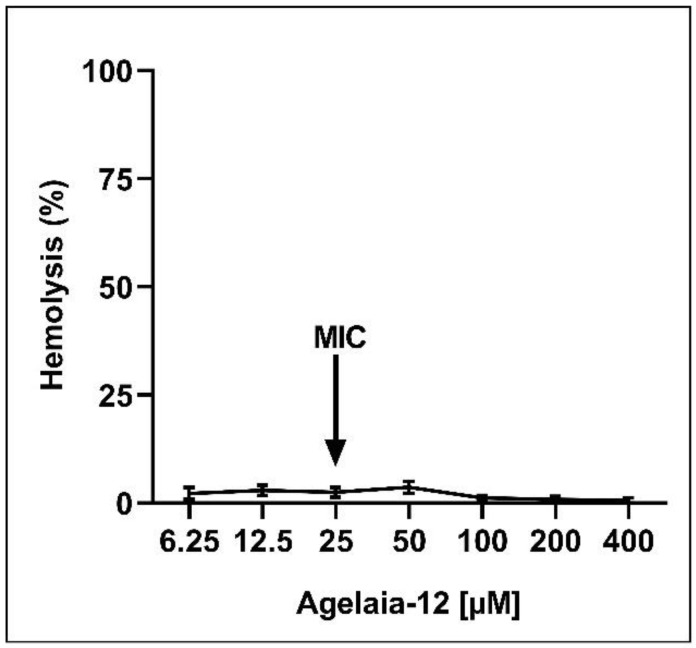
Hemolysis assay. Percentage of human red blood cell hemolysis after incubation with different concentrations of Agelaia-12. Supernatants were evaluated at OD 540 nm. The arrow indicates the MIC of Agelaia-12. Triton-X 1% was used as a hemolysis positive control.

The potential use of a drug depends on its low toxicity to the host combined with a high antimicrobial action [53]. Thus, the level of cytotoxicity of Agelaia-12 in red blood cells was investigated by the hemolysis assay. The hemolysis assay with concentrations 16 times higher than the MIC did not result in considerable toxicity (Figure 4). It was not possible to determine the therapeutic index within the tested concentrations.

### 3.5. Agelaia-12 Controls the Intracellular Bacterial Growth of Infected Macrophages

**Figure 5:**
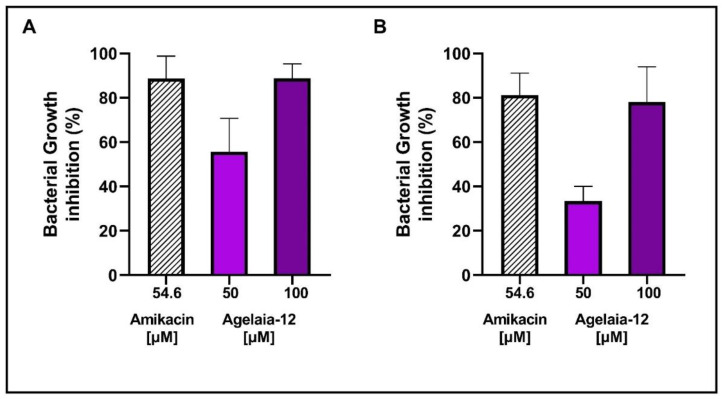
Percentage of bacterial growth inhibition of *M. abscessus* or *M. massiliense* in infected macrophages. After 3 h of infection for bacterial internalization, *M. abscessus* (**A**) or *M. massiliense* (**B**), macrophages were treated with amikacin (54.6 μM) or Agelaia-12 for 24 h. After discarding the supernatant, macrophages were lysed and the bacterial load determined. The percentage of bacterial growth inhibition was determined in comparison to nontreated infected macrophages.

Macrophages are important cells in the innate immune response and natural hosts of pathogens, including mycobacteria [54]. Thus, macrophages were infected with *M. abscessus* or *M. massiliense* and treated with Agelaia-12 or amikacin. Figure 5 shows that treatment with Agelaia-12 inhibits bacterial growth at concentrations of 50 and 100 μM. The best inhibition was obtained when Agelaia-12 was used at 100 μM and was similar to amikacin.

### 3.6. Treatment of M. abscessus- or M. massiliense-Infected Mice with Agelaia-12 Reduces the Lung Bacterial Load and Lung Inflammation

**Figure 6:**
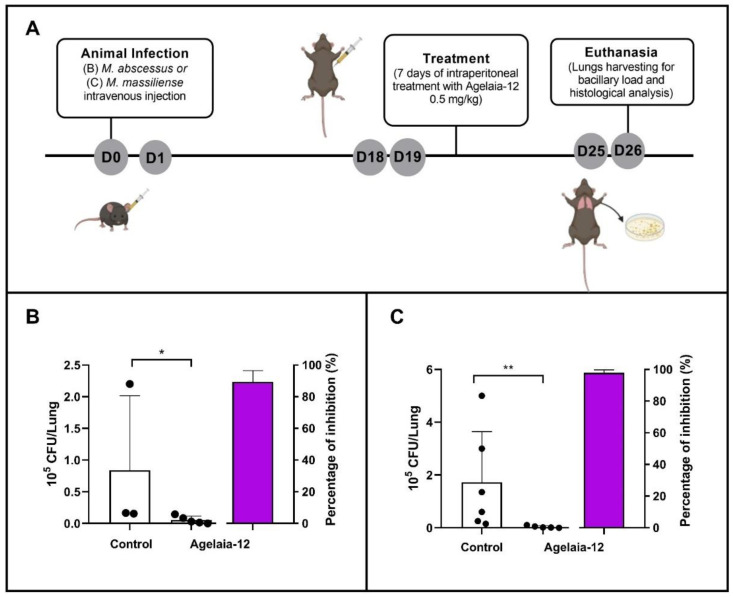
In vivo assay for evaluation of Agelaia-12 treatment of IFN-y KO infected mice. (**A**) Timeline of the in vivo assay conducted with female and male IFN-y KO mice (day zero: infection of the animals with 10^6^ CFU/mL of *M. abscessus* or 10^8^ CFU/mL of *M. massiliense* intravenously). On day nineteen (D19), animals were treated with PBS (control group) or with Agelaia-12 intraperitoneally for seven days. On day twenty-six (D26), animals were euthanized for lung collection and analysis of the bacterial load and histological studies. (**B**,**C**) Mycobacterial load in the lungs of the control group and those treated with Agelaia-12 with *M. abscessus* ATCC (**B**) or *M. massiliense* GO06 (**C**). The far right column in each graph corresponds to the growth inhibition percentage by Agelaia-12 treatment. * Significant differences (*p* < 0.05); ** significant differences (*p* < 0.001).

To evaluate the ability of Agelaia-12 to treat mycobacterial infection, IFN-y KO mice were infected with *M. abscessus* or *M. massiliense* and treated according to the timeline described in Figure 6A. The treatment of animals infected with Agelaia-12 resulted in a significant reduction in the pulmonary mycobacterial load compared to the animals in the control group, for both strains evaluated (Figure 6B,C).

**Figure 7:**
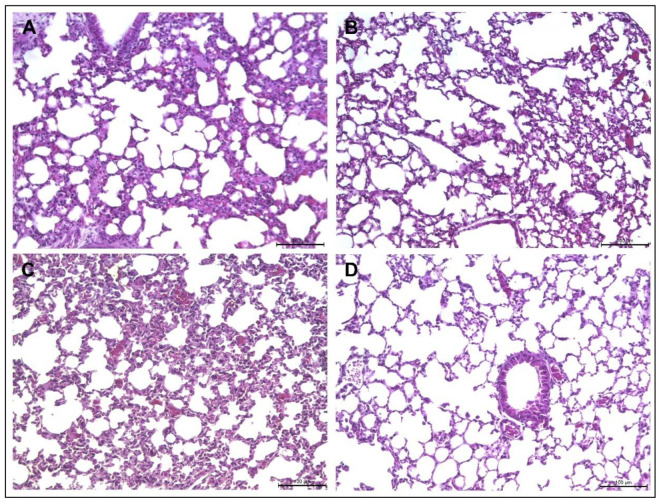
Lung histology of IFN-y KO mice infected with *M. abscessus* or *M. massiliense* and treated with Agelaia-12. *M. abscessus* (**A**) or *M. massiliense* (**C**) infected animals presented difuse inflammatory lesions. After treatment with Agelaia-12, mice infected with *M. abscessus* (**B**) showed visually less inflammatory lesions than nontreated mice. Siimilarly, mice infected with *M. massiliense* and treated with Agelaia-12 showed visually less inflammatory lesions (**D**) than nontreated mice.

Because the treatment with Agelaia-12 reduced the bacterial load, the lung lesions caused by infection were evaluated. As observed in Figure 7A, an intense diffuse inflammatory lesion without the formation of a defined granuloma was seen, which appeared to be reduced after Agelaia-12 treatment (Figure 7B). Similarly, in animals infected with *M. massiliense,* diffuse inflammatory lesions were observed (Figure 7C); however, the inflammatory lesions appear to be resolved after treatment with Agelaia-12 (Figure 7D). Due to the diffuse inflammatory lesions, it was not possible to quantify the area of lesion in the lung.

## 4. Discussion

Wasp venoms are synthesized as a defense strategy against predators and have several constituents, such as enzymes, peptides, and volatile and bioactive compounds [55]. Peptides stand out for having antibacterial and cytotoxic activities and their investigation is crucial for developing new drugs for therapy against multidrug-resistant microorganisms. In this study, we evaluated Agelaia-12, which is an AMP bioinspired from Agelaia-MPI, isolated from social wasp *Parachartergus fraternus* venom, and here we show for the first time its antibacterial effects against *M. abscessus* and *M. massiliense*.

The peptide Agelaia-12 has a conserved polar region consisting of four lysine residues, an apolar region with three isoleucine, one tryptophan, two leucine, and one glycine residues, and an anionic region with an aspartate residue (Figure 1), where the insertion of two lysine residues contributed to its amphipaticity and alpha helix conformation, which might favor cell penetration [56]. Alpha helix AMPs are the most abundant in nature, widely found in insects, usually rich in lysine, glycine, and leucine amino acids, and often responsible for interaction with target membranes [57]. This interaction occurs because alpha helix AMP is rich in positively charged amino acid residues, which can electrostatically bind to negatively charged membranes and favor pore formation, destabilizing them, which can lead to cell death [58].

Since alpha helix AMP has been widely evaluated as an important molecule in inhibiting bacterial growth, an in vitro test was carried out in which the action of Agelaia-12 against *M. abscessus* and *M. massiliense* was observed and an MIC of 25 μM was determined (Figure 2A,B). These findings corroborate previous studies which also demonstrated that AMPs inhibited the growth of mycobacteria, as observed in the work of [38], in which it was found that the ToAP2 AMP, which has two alpha helices, was able to present bactericidal activity against *M. massiliense* at 200 μM. A similar result was observed for the NDBP-5.5 peptide in the study by [37], a neutrally charged peptide with amphipathic properties. The modifications that resulted in the bioinspired Agelaia-12 increased its overall cationic charge to +3, which may have contributed to the change from a bactericidal profile, as observed in Agelaia MPI against *A. baumannii* [59], to a bacteriostatic action, as observed here.

Generally, the mechanism of action of AMPs occurs through their electrostatic interaction with the outer membrane of the microorganisms, causing modifications, i.e., forming a cylindrical pore as in the barrel model or forming a channel in the toroidal model or forming carpets [60,61], all of which result in envelope disfunction. Studies demonstrated that AMPs could promote structural changes in the wall of mycobacteria [39], resulting in cell surface/permeability modifications. Although MEV analysis of the action of Agelaia-12 on *M. massiliense* did not show morphological changes characteristic of pore formation, a significant change in the cellular aggregation and filament formation was observed. The morphological changes may also be related to the capacity of these bacteria to form biofilms [62], and thus the influence of Agelaia-12 on biofilm formation should be investigated in future studies. The cell surface was affected by the action of Agelaia-12, as shown with greater Congo red dye retention, in similar extension as under polymyxin action. It is interesting to note that polymyxin B has a cationic overall charge and its mechanism of action is by forming aggregates and causing dysfunction of membrane proteins [63,64]. Although polymyxin acts mainly on Gram-negative bacteria, its antimicrobial activity against other classes of Gram-positive bacteria was shown here and in other works [63].

Hydrophobicity is an important characteristic that determines the biological activity of antimicrobial peptides, in which the higher the hydrophobicity, the greater the binding of AMP to membranes, the higher the cytolytic activity, and the lower the solubility [65]. Most mastoparans synthesized by wasps have high hemolytic action, which is highly correlated with the hydrophobicity of the AMP molecule, compromising their use as antimicrobials. Thus, modifying mastoparans’ hydrophobicity will aggregate a desired value to mastoparans [66]. Agelaia-12 presented negligent hemolysis (5%), even at a concentration 16 times higher than the MIC, unlike Agelaia-MPI, which presented approximately 70% of hemolysis at a concentration of 0.1 μM [67]. This difference might be due to the sequence shortening, with removal of the nonpolar hydrophobic residue’s alanine and leucine [68].

Mycobacteria reside inside endocytic vesicles during infection, frequently modulating the pH within these vesicles and evading the hosts’ mechanisms of defense [69,70]. In order to verify if Agelaia-12 would retain its antimicrobial properties within this environment, macrophages were infected and treated with Agelaia-12. Agelaia-12 was able to significantly reduce the mycobacterial load of macrophages infected with *M. abscessus* or *M. massiliense*, equivalent to that observed with the amikacin, showing its ability to overcome the eukaryotic tolerance mechanisms, such as macrophage efflux pumps [70,71]. Macrophages treated with Agelaia-12 remained viable, corroborating with the low toxicity shown for erythrocytes.

The MAC is an important group of microorganisms that can cause respiratory infections, which lead to a decline in lung function [72], with nontuberculous lung disease being one of the most common infections, especially in individuals with impaired immunity or with comorbidities [14,73]. Agelaia-12 inhibited 98% of the lung bacterial load of mice infected with *M. abscessus* or *M. massiliense*, which reinforces the antimycobacterial action observed for macrophage cultures. AMPs may be susceptible to destruction by host proteases or other mechanisms [74]. Although we have not investigated the integrity of Agelaia-12 during the treatment of mice, the effective reduction in the bacterial load shows that it remained active within the infected animals. The low mycobacterial load was corroborated by the histopathological findings, where a visual reduction in the diffuse inflammatory lesions was observed within AMP-treated mice. Altogether, Agelaia-12 was able to treat MAC infection, reducing the inflammatory response with minimal toxicity.

Despite the potential therapeutic benefits, there are some limitations in the clinical application of AMPs, such as stability, cytotoxicity, and bioavailability [75], as well as degradation by serum proteolytic enzymes and their biodistribution in the body [74]. However, the evolving possibility of AMPs’ chemical synthesis and their bioactivity assays will boost the expansion of the clinical application of these molecules.

## 5. Conclusions

The antimicrobial peptide Agelaia-12 showed promising results against fast-growing mycobacteria from the *M. abscessus* complex, reducing 98% of the bacterial load in the lungs of infected mice with low toxicity for human blood cells, suggesting it can be an alternative drug for the treatment of mycobacteriosis.

## 6. Patents

The novel synthetic sequence of peptide Agelaia-12 is originally described by the patent coded BR102020022526-0 from Junqueira-Kipnis A.P. et al. [45] and issued by the Brazilian National Institute of Intellectual Property (INPI).

## Figures and Tables

**Table 1:** Physicochemical properties of AMP Agelaia-12.

Characteristics	
Molecular formula	C71H123N17O15
Molecular mass	1454.86
Net charge	+3
Isoelectric point	10.00
Extinction coefficient	5500 M^−1^ × cm^−1^
Hydrophobicity	+15.94 Kcal × mol^−1^

## Data Availability

The raw data supporting the conclusions of this article will be made available by the authors on request.
